# Current insights into hormonal regulation of microspore embryogenesis

**DOI:** 10.3389/fpls.2015.00424

**Published:** 2015-06-10

**Authors:** Iwona Żur, Ewa Dubas, Monika Krzewska, Franciszek Janowiak

**Affiliations:** The Franciszek Górski Institute of Plant Physiology, Polish Academy of SciencesKraków, Poland

**Keywords:** crop species, hormonal regulation, microspore embryogenesis, plant growth regulators, phytohormone crosstalk

## Abstract

Plant growth regulator (PGR) crosstalk and interaction with the plant’s genotype and environmental factors play a crucial role in microspore embryogenesis (ME), controlling microspore-derived embryo differentiation and development as well as haploid/doubled haploid plant regeneration. The complexity of the PGR network which could exist at the level of biosynthesis, distribution, gene expression or signaling pathways, renders the creation of an integrated model of ME-control crosstalk impossible at present. However, the analysis of the published data together with the results received recently with the use of modern analytical techniques brings new insights into hormonal regulation of this process. This review presents a short historical overview of the most important milestones in the recognition of hormonal requirements for effective ME in the most important crop plant species and complements it with new concepts that evolved over the last decade of ME studies.

## Introduction

Plant growth regulators are known as key signaling molecules controlling plant growth and development, and initiating signal transduction pathways in response to environmental stimuli (Kohli et al., 2013). The role played by PGRs in ME has been examined widely but usually using the traditional ‘one-factor-at-a-time’ and ‘trial-and-error’ techniques. Hormonal requirements determined through such empirical methods were usually optimized for particular cultivars or genotypes. Once identified, positively acting combinations of PGRs were usually used standardly for years with small modifications introduced in the case of less responsive genotypes. Only in a few cases endogenous levels of PGRs were analyzed and taken into consideration in studies examining their influence on ME effectiveness (Dollmantel and Reinert, 1980; Delalonde and Coumans, 1998; Gorbunova et al., 2001; Lulsdorf et al., 2012). Moreover, usually only one or two groups of phytohormones were analyzed whereas there is growing evidence indicating that – as could be expected – it is the complex PGR crosstalk and its interaction with the plant’s genotype and environmental factors which controls the initiation and the course of the process. The complexity of the PGR network which could exist at the level of biosynthesis, distribution, gene expression, or signaling pathways, renders the creation of an integrated model of ME-control crosstalk impossible at present. However, the analysis of the published data together with the results received recently with the use of modern analytical techniques bring new insights into hormonal regulation of this process. New concepts that evolved over the last decade of ME studies together with a short historical review showing the most important milestones in the recognition of hormonal requirements for effective ME are presented below. The review concerns the most important crop plants, both model species, and species well-known for their recalcitrance to most *in vitro* approaches like oat, rye, grain legumes, and cassava (Supplementary Table S1).

## Auxins and Cytokinins

Particularly important for *in vitro* cultures is the concerted action of auxins and cytokinins which control cell division and morphogenesis. These two hormone groups usually act antagonistically but their effects are modulated by plant genome and tissue specificity (Moubayidin et al., 2009). Various combinations of auxins and cytokinins have been used in media designated for *in vitro* anther culture, whereas in the majority of isolated microspore cultures exogenous PGRs were not required for ME initiation (for details see Supplementary Table S1). For several plant species instead of exogenous PGRs, co-culture with so-called immature ‘ovaries’ (accurately, pistils) is critical to sustain microspore-derived embryo development (Hul and Kasha, 1997; Li and Devaux, 2001; Zheng et al., 2002; Lantos et al., 2009). Similar or even better results could be received through the use of conditioned medium, prepared by culturing isolated ‘ovaries’ or microspores of responsive plant genotypes. In isolated microspore culture of recalcitrant wheat cultivars (Zheng et al., 2002) live ‘ovary’ co-culture alone was not effective, while the addition of medium preconditioned by ‘ovaries’ increased the yield of microspore-derived embryos more than 100-fold. Similarly, conditioned medium extracted from actively growing microspores of barley broke the recalcitrancy of isolated oat microspores and resulted in regeneration of fertile green plants (Sidhu and Davies, 2009). Despite many attempts, the effect of ‘ovary’ co-culture could not be successfully substituted by any treatment or any exogenously applied substance. It is supposed that the ‘ovaries’ are a sources of active signaling molecules that increase microspore-derived embryo yield and improve green plant regeneration. The involvement of auxin-like substances and/or arabinogalactans (Baldwin et al., 1993; Borderies et al., 2004; Letarte et al., 2006) has been postulated, but the mechanism of their influence remains unexplained.

Among auxins, the first and most widely used for ME initiation was IAA (Guha and Maheshwari, 1964). It is an essential phytohormone ubiquitous throughout the plant kingdom and involved in the regulation of a wide spectrum of physiological processes (Davies, 2010). Later on it was frequently replaced by more stable synthetic auxin analogs: 2,4-D, NAA, DIC or PIC and its combinations (for details see Supplementary Table S1; **Table 1**). 2,4-D is one of the most often used culture media supplements, applicable for both dicotyledonous and monocotyledonous plants (Raghavan, 2004). Its high effectiveness in the induction and maintenance of callus and suspension cultures from somatic tissues resulted in its application as ME stimulus. Other synthetic auxin analogs were also first tested in somatic tissue cultures as inducers of embryogenesis (PIC, DIC) or organogenesis (NAA; Gaspar et al., 1996). Natural auxins: IBA and PAA are used less frequently (for details see Supplementary Table S1). For decades, IBA has been used commercially for plant propagation, being more effective than IAA in stimulation of adventitious root formation. Its effectiveness can be at least partially explained by its higher stability and lower predisposition to the formation of inactive conjugates. Its possible direct involvement in ME initiation has been postulated recently (Dubas et al., 2013a). Similarly, PAA was applied mainly for stimulation of plant regeneration, its beneficial effects on androgenic plant production having been reported for wheat and barley (Ziauddin et al., 1992). In addition to acting as an active auxin, PAA may inhibit polar auxin transport (Morris and Johnson, 1987), regulating the level of free IAA. In plants, it is present at levels 10- to 100-fold lower in comparison with IAA. Due to its low activity (Normanly et al., 2010), it is usually supplemented in much higher concentrations to media used for ME (**Table 1**).

**Table 1:** The most popular PGRs and their concentration ranges [mg l^-1^] used standardly in media dedicated for ME induction and microspore-derived embryo regeneration.

PGRs	Induction media	Regeneration media
**Auxins and synthetic auxin analogs**		
IAA	1–4	0.01–3.5
IBA	0.5–1	1–2
PAA	1–100	1–4
2,4-D	0.1–8	0.5–3
NAA	0.5–2.5	0.05–5
Dicamba	0.1–2.5	-
Picloram	0.07–4	-
**Anti-auxin and auxin transport inhibitors**		
PCIB	1–5	-
TIBA	0.05–2	0.1–1
**Cytokinins**		
BAP	0.05–3	0.1–5
Kinetin	1	0.1–5
Z/ZR	0.1–1	0.5–2.2
TDZ	0.1–1	-
2iP	0.0001–0.4	0.1
**Other PGRs**		
GA_3_	0.001–5	0.01–0.1
ABA	0.001–10	0.05–3

*For more details see review in Supplementary Table S1*.

Interestingly, besides auxins, several inhibitors of auxin biosynthesis (AZI) or auxin polar transport TIBA, NPA, or 1-NOA as well as anti-auxins OCPIB, PCIB have been used quite frequently (for details see Supplementary Table S1; **Table 1**). All these chemicals influence embryo development through disruption of auxin homeostasis (Lankova et al., 2010). However, depending on the plant species, type of substance and procedures used, the results were ambiguous or even contradictory. For example, preculture of *Nicotiana tabacum* anthers in the presence of the inhibitor of AZI and anti-auxin (OCPIB) resulted in enhanced plantlet formation (Dollmantel and Reinert, 1980). In contrast, IAA-oxidase activator, CPIBA, IAA transport inhibitor (quercetin), and IAA-oxidase inhibitor (dopamine) did not give positive results in maize anther cultures (Delalonde and Coumans, 1998). Due to structural similarity, anti-auxins (Jönsson, 1961) can compete with IAA at the binding site of its receptors (McRae and Bonner, 1953) and exhibit some antagonistic effects. Lower concentration of PCIB (20 μM) enhanced the development of microspore-derived embryos of *Brassica juncea* and *B. napus*, while higher doses were detrimental and resulted in a high frequency of morphologically abnormal embryo formation (Agarwal et al., 2006; Ahmadi et al., 2012). In *B. rapa*, critical ME-stimulating concentration of PCIB was twofold higher (40 μM), but similarly its overdose decreased microspore-derived embryo yield and increased the frequency of morphological abnormalities (Zhang et al., 2011). The effect of TIBA (1 μM), which conjugates specifically to the ingression site and inhibits polar transport of IAA, on barley ME was highly genotype-dependent (Cistué et al., 1999). Although it decreased the number of dividing microspores in some cultivars, a tendency to produce a higher percentage of embryos and to improve embryo quality was also observed. Higher doses (2–4 μM) of TIBA were beneficial for low responsive cultivar, increasing well developed embryo production and reducing albinism. TIBA could also affect later phases of microspore-derived embryo development. The treatment applied to *B. napus* cv. Topas at the preglobular/globular stages of embryo development resulted in altered shoot apical meristem development and in production of one fused cotyledon, which indicates a continuation of radial symmetry (Ramesar-Fortner and Yeung, 2006). However, tillers pre-treatment with 5 μM PCIB or 10 μM TIBA had no effect on ME induction in triticale anther culture (Żur et al., 2015). Similar to TIBA in the report of Cistué et al. (1999), PCIB stimulated plant regeneration but only in the highly recalcitrant triticale genotype. The supplementation of ME-induction medium with the same concentrations of TIBA or PCIB did not improve the efficiency of the process in the case of the recalcitrant genotype and significantly decreased the number of microspore-derived embryos produced by the responsive one (Żur et al., 2015).

The negative effect of NPA on embryogenesis was observed in microspore suspension of oak (Rodriguez-Sanz et al., 2014). This compound together with 1-NOA are potent synthetic auxin inhibitors (Lankova et al., 2010). It was proved that NPA strongly inhibits auxin eﬄux (Petrasek et al., 2003), whereas 1-NOA blocks both auxin influx and eﬄux. NPA interferes with actin dynamics being under the control of auxin itself, while 1-NOA action has been suggested to be related to the dynamics of membrane vesicle transporting auxin carriers (Titapiwatanakun and Murphy, 2009; Lankova et al., 2010).

Two major properties of cytokinins that predispose these adenine derivatives for *in vitro* cultures are their abilities to induce cell division and differentiation. Their effects result from co-action with auxins, but each of these PGR groups seems to control different phases of the cell cycle: auxins – DNA replication, whereas cytokinins – mitosis and cytokinesis (Gaspar et al., 1996). Among cytokinins, kinetin (N6-furfuryladenine; KN), 6-benzylaminopurine (BAP), and zeatin (Z) have been frequently tested both in ME induction and regeneration media (for details see Supplementary Table S1; **Table 1**). Other kinds of cytokinins, like thidiazuron (*N*-phenyl-*N*’-1,2,3-thiadiazol-5-ylurea; TDZ), N6-(2-isopentenyl)-adenine (2iP) or meta-topoline (6-3-hydroxybenzylaminopurine, mT) are less popular ingredients of culture media (Kumar et al., 2003; Grewal et al., 2009; Esteves et al., 2014). TDZ has been used successfully *in vitro* to induce adventitious shoot formation and to promote axillary shoot proliferation. It is particularly effective with recalcitrant woody species. However, prolonged exposure to this cytokinin may cause problems such as hyperhydricity and abnormal shoot or root development (Lu, 1993). 2iP was used for ME initiation in anther culture of *Cicer arietium* (Grewal et al., 2009), whereas regeneration medium supplemented with mT proved beneficial for green plants production in microspore culture of barley (Esteves et al., 2014).

## Abscisic Acid

Besides auxins and cytokinins, ABA, known as a ubiquitous plant stress hormone, has been claimed to play a role in ME-inducing signal transduction system (Maraschin et al., 2005; Żur et al., 2012, 2015; Dubas et al., 2013b; Ahmadi et al., 2014). It is well documented that plant cells and tissues usually increase their ABA level in response to different biotic and abiotic stresses (Zeevaart and Creelman, 1988; Christmann et al., 2004; Cutler et al., 2010). During ME induction various stress conditions (e.g., starvation, cold, osmotic stresses) have been commonly used as a trigger of microspore switch toward sporophytic development pathway (Touraev et al., 1997; Zoriniants et al., 2005). ABA level increases in tissues and microspores exposed to these stresses and many reports have suggested a causal involvement of ABA in ME induction, describing the positive influence of ABA accumulation on the effectiveness of this process (Reynolds and Crawford, 1996; van Bergen et al., 1999; Wang et al., 1999; Żur et al., 2008, 2012). Furthermore, a positive relationship has been shown to exist between higher regeneration efficiency and increased endogenous ABA level during ME induction by osmotic stress in barley (Hoekstra et al., 1997; van Bergen et al., 1999). These positive ABA effects on ME have been confirmed in several manipulative experiments with a treatment with exogenous ABA or its inhibitor fluridone (Imamura and Harada, 1980; Reynolds and Crawford, 1996; Wang et al., 1999; Guzman and Arias, 2000). In *Hordeum* species the addition of 10^-7^ M ABA enhanced the regeneration of plants at sub-optimal anther pre-treatment conditions, while ABA-biosynthesis inhibitor fluridone strongly reduced regeneration efficiency, particularly green plant production (Hoekstra et al., 1997; van Bergen et al., 1999). In another study of rapeseed, ME efficiency was improved by exogenous ABA treatment – 0.5 mg ABA l^-1^ for 12 h enhanced ME threefold compared with untreated cultures and increased normal plantlet regeneration by 68% (Ahmadi et al., 2014). In turn Żur et al. (2008, 2012) observed a significant endogenous ABA increase during ME induction by low temperature stress in triticale. However, there was no linear relationship between the extent of ABA accumulation and ME efficiency in the population of 72 triticale DH lines (Żur et al., 2012). On the contrary, higher level of endogenous ABA significantly diminished green plant regeneration efficiency. Therefore, it seems that the induction of ME requires a certain genotype-specific threshold level of ABA, which initiates a signaling cascade switching the program of embryogenic development. Moreover, it seems that a specific PGRs homeostasis and auxins/cytokinins/ABA crosstalk is a more important prerequisite for effective ME than the level of individual PGRs (Żur et al., 2015). It has also been discovered that microspores’ membrane fluidity may indirectly affect the level of ABA accumulation within the cell (Dubas et al., 2013b). Those findings verified the hypothesis about the influence of ABA on ME induction in rapeseed and pointed out that increased ABA concentration (to about 2.1 μM) in heat-treated microspores enhanced ME. Altogether, the role of ABA in microspore reprogramming is complex – it acts as a common anti-stress factor increasing microspores’ viability during ME induction, and on the other hand, ABA-induced signaling cascade plays a vital role in the activation of many genes (mainly controlling the synthesis of LEA proteins), in the activity of enzymes and in the ox-redox status as well as interacts with other PGRs (Maraschin et al., 2005; Żur et al., 2012, 2014, 2015; Ahmadi et al., 2014).

## Other Plant Growth Regulators

Despite many studies on microspore and anther culture in crop species, the effects of phytohormones such as gibberellins (GAs), brassinosteroids (BRs), jasmonic acid (JA), salicylic acid (SA), or ethylene on ME are not fully recognized.

GAs are involved in a wide range of developmental responses (Moshkov et al., 2008). They are required for normal pollen, anther and seed development, and are probably involved in a broad spectrum of responses to abiotic stress (Colebrook et al., 2014), but a complete understanding of their specific function remains elusive (Swain and Singh, 2005). Only scarce information is available on GAs in cultured cells. Plant tissue cultures can generally be induced to grow and differentiate without GAs. One of the most bioactive forms, GA_3_ is generally used in plant tissue to stimulate stem elongation. It was also supposed to be an essential ingredient of media for culturing cells at low densities (Stuart and Street, 1971). In microspore cultures of *B. napus* and *Solanum tuberosum*, GA_3_ improved plantlet regeneration (Supplementary Table S1), mainly *via* elongation of the embryo axis and acceleration of its maturation (Haddadi et al., 2008). Similarly Ahmadi et al. (2012) reported that the highest percentage of normal *B. napus* plantlet regeneration (40%) was received as a result of 0.05–0.1 mg l^-1^ GA_3_ treatment. More attention has been attracted by a wide range of synthetic substances, called ‘anti-gibberellins,’ which block GAs biosynthetic pathways. In the studies of Biddington et al. (1992), the addition of paclobutrazol into induction media inhibited embryo production in anther cultures of Brussels sprout. However, the authors suggested that this effect could be caused not only by inhibition of GA-biosynthesis but also by inhibition of sterol biosynthesis. Another inhibitor of GA-biosynthesis, uniconazole, applied to *B*. *napus* embryo at the globular stage of development significantly reduced axis elongation (Hays et al., 2002).

Brassinosteroids (BRs) are a class of plant steroidal hormones that regulate multiple developmental and physiological processes essential for plant growth and development. Their involvement in cell elongation and division, vascular differentiation, senescence, flowering time, male fertility, pollen development, seed size, photomorphogenesis, and resistance to biotic and abiotic stresses has been reported (Clouse et al., 1996; Li and Chory, 1999; Ye et al., 2010; Clouse, 2011). BRs, in particular 24-epibrassinolide (EBr), increased frequency of induction of both somatic (Azpeitia et al., 2003; Pullman et al., 2003) and ME (Ferrie et al., 2005; Malik et al., 2008). The role of EBr may be related to protection against abiotic stresses as its positive impact on the acquisition of thermotolerance was reported (Divi et al., 2010). Other BR, brassinolide (BL) also enhanced embryogenesis and the quality of microspore-derived embryos in *B. napus* and *B. juncea* (Ferrie et al., 2005; Belmonte et al., 2010). The addition of BRs did not affect plant regeneration but seems to influence chromosome doubling. Moreover, depletion of cellular BL decreases microspore-derived embryo production and disrupts the architecture of the apical meristems of *B. napus* (Belmonte et al., 2010).

As ME is induced by stress treatment it could be supposed that not only ABA, but also other stress hormones like jasmonic acid (JA), salicylic acid (SA), or ethylene can be involved in this process.

JA is widely distributed in the plant kingdom and regulates a wide range of processes from growth and photosynthesis to reproductive development. The most important is the role connected with plant defense reactions against biotic and abiotic stresses (Santino et al., 2013). In anther cultures of barley, ME-induction treatment resulted in higher expression of three genes encoding enzymes involved in JA biosynthesis (Jacquard et al., 2009). Ahmadi et al. (2014) claimed that the supplementation of induction medium with 1.0 mg l^-1^ JA for 24 h improved embryo yield in microspore cultures of *B. napus*. Moreover, the addition of 0.5 mg l^-1^ JA for 12 h resulted in better plantlet regeneration.

SA, a plant phenolic derivative, is now considered to be a hormone-like endogenous regulator and its role in the defense mechanisms against biotic and abiotic stress is well documented (Catinot et al., 2008). Being a mobile molecule, SA is capable of acting as a cell signal that senses, amplifies, and transmits information initiating the embryogenic program (Mulgund et al., 2012). There are several papers that describe the application of SA to culture media in order to improve ME efficiency. In the above-mentioned work, Ahmadi et al. (2014) reported a positive effect of short-term application (6 h) of 0.2 and 0.5 mg l^-1^ of SA on *B. napus* microspore-derived embryo yield. The mechanism of SA action could be connected with its ability to increase the activity of superoxide dismutase (H_2_O_2_-producing enzyme), and to inhibit ascorbate peroxidase and catalase activities (H_2_O_2_-decomposing enzymes), thus leading to endogenous H_2_O_2_ accumulation, which is supposed to initiate ME (Larqué-Saavedra, 1978, 1979; Leslie and Romani, 1988; Luo et al., 2001; Żur et al., 2014).

Ethylene is a gaseous plant hormone involved in many developmental processes – seed germination, root development, flower senescence, abscission, and fruit ripening (Kumar et al., 2009). Its biosynthesis is tightly regulated by internal signals and environmental stresses, like wounding, low temperature, hypoxia, or pathogen attack (Wang et al., 2002). Its role in *in vitro* callus growth, organo- and embryogenesis has been suggested (Kumar et al., 2009). It was reported that embryogenesis in barley can be stimulated by both promoters and antagonists of ethylene, depending on the genotype (Cho and Kasha, 1989). It suggests that the response depends upon how much ethylene is being produced and that an optimum level of ethylene is required for ME initiation. More often, positive effects induced by substances known as inhibitors of ethylene action – silver nitrate (Prem et al., 2005), activated charcoal (Prem et al., 2008), AVG or cobalt chloride (Leroux et al., 2009) were observed. On the other hand, there is also evidence reporting benefits from ethylene precursor ACC or promoter ETP. Their application increased ME initiation in anther culture of barley (Evans and Batty, 1994) and oat (Kiviharju et al., 2005).

## New Concepts Describing PGRs Involvement in ME Regulation

### New Kinds of PGRs Possibly Involved in ME Regulation

Although IBA is commonly considered to be only an IAA precursor and storage form (Woodward and Bartel, 2005; Korasick et al., 2013), some evidence suggests that it could act directly as an active auxin (Ludwig-Muller, 2000; Poupart and Waddell, 2000; Zolman et al., 2000). Recent results shown by Dubas et al. (2013a) suggest that the increased level of IBA in *B. napus* microspores under heat shock treatment might be used as a marker of cell embryogenic competence. However, IBA accumulation was not sufficient for ME initiation in the case of recalcitrant genotypes. Similar results were received in anther cultures of triticale (Żur et al., 2015), where higher concentration of IBA seems to be advantageous for effective ME induction. However, because IBA pool in triticale anthers comprises only about 1% of the total auxins content, it is questionable whether differences in its concentration are of any significance. In the same report, *trans* and *cis* isomers of *t*Z, *c*Z and *t*ZR, *c*ZR were detected in anthers of eight DH lines of triticale. Interestingly, *c*Z commonly regarded as cytokinin derivative without any or with low biological activity, prevailed significantly and positively correlated with ME induction. Similarly, in reports of Emery et al. (1998), Vyroubalová et al. (2009), and Kudo et al. (2012) *c*Z appeared to be the dominant form of cytokinins in specific plant organs and/or stages of development. In a recently published report, Gajdošová et al. (2011) concluded that *c*Z can be qualified as a regulator of cytokinin responses in plants under growth-limiting conditions. Another finding of Żur et al. (2015) was a relatively high concentration of KN-like compound and its negative correlation with ME efficiency. KN was the first compound identified as cytokinin, but for many years it was classified as a product of DNA rearrangement not produced by plant cells. This opinion started to change in the last decade, as sources of KN in biological samples were found in cellular DNA, plant tissues and extracts (Barciszewski et al., 1996, 1999, 2007; Ge et al., 2005). The role of endogenous KN and the molecular mechanisms of its action are not well understood, although some data indicate its strong antioxidant properties and some ABA- and JA-antagonistic effects (Barciszewski et al., 2000).

### High Concentrations of PGRs as a Stress Factor

Higher concentration (5–10 mg l^-1^) of 2,4-D stimulated ME initiation in some recalcitrant plant species, namely oat (Kiviharju and Tauriainen, 1999), *Triticum turgidum* (Jauhar, 2003), and cassava (Perera et al., 2014). It has been suggested that 2,4-D is not only an auxin analog but at higher concentrations acts as a stress factor effectively triggering embryogenic pathway of cell development (Gaj, 2004). The observed effect is probably the result of concerted PGRs action as evidence that 2,4-D regulates the activity of genes associated with auxin, ABA and ethylene biosynthesis has been reported (Raghavan et al., 2006). Short-term treatment with extremely high concentration of this substance (15–45 mg l^-1^ 2,4-D for 15–45 min) has been recently used as an effective substitute of classical heat shock treatment (Ardebili et al., 2011) for *B. napus* ME initiation. It has been proposed as an alternative for plant species whose microspores are extremely sensitive to classical stresses.

### Endogenous Level of PGRs and their Interaction with their Exogenously Applied Analogs

Inconsistent or even contradictory effects of various PGRs and their inhibitors suggest that the endogenous level of natural phytohormones and its balance with exogenously applied ones can be crucial both for yield and quality of microspore-derived embryos.

The first report pointing out that endogenous auxins level can determine anther culture responsiveness was published as early as by Dollmantel and Reinert (1980). Next, the results published by Gorbunova et al. (2001) indicated that wheat genotypes with high endogenous IAA content required lower concentration of 2,4-D in induction medium. Other data showing that anti-auxin (PCIB) and auxin transport inhibitor (TIBA) can stimulate microspore-derived embryo formation, probably due to overcoming the inhibitory effect of high auxin concentration, were published by Cistué et al. (1999) and Agarwal et al. (2006). Also in the case of triticale (Żur et al., 2015), for which ME-induction medium was supplemented with 1 mg l^-1^ DIC, 1 mg l^-1^ PIC, and 0.5 mg l^-1^ KN, anther cultures of responsive DH lines were characterized by significantly lower endogenous/exogenous auxins ratio in comparison to recalcitrant genotypes. In the same cultures, higher embryogenic potential was associated with significantly higher endogenous/exogenous cytokinins ratio.

Generally, exogenous PGRs are not required for ME in *Brassica* species. However, too low levels of endogenous auxins and/or cytokinins could disturb the proper course of the process, especially the transition from the radial to the bilateral microspore-derived embryo symmetry, as it was observed in *B. napus* (Ramesar-Fortner and Yeung, 2006). Similarly, the addition of 0.1–0.3 mg l^-1^ BAP significantly improved microspore-derived embryo yield in several *B. rapa* subspecies (Takahashi et al., 2012). Recently, Prem et al. (2012) and Dubas et al. (2014) showed how endogenous auxin distribution influenced embryo development in microspore suspension of *B. napus*. Precise endogenous auxin estimation in transgenic DR5rev::GFP or DR5::GUS microspores of highly embryogenic spring rape line (Dubas et al., 2014) revealed IAA concentration at 1.01 μM in microspores under ME-initiating heat treatment (1 day at 32°C). It could be supposed that such IAA concentration is optimal for further embryo development.

### Crosstalk of Various PGRs and their Interaction with Stress Treatment and Plant Genotype in ME Initiation

A number of reviews highlighting phytohormone crosstalk in plant growth, development and response to abiotic and biotic stresses have been published recently (Depuydt and Hardtke, 2011; Hou et al., 2013; Kohli et al., 2013; Lyons et al., 2013; Wang et al., 2013). It is also well known that altered homeostasis of PGRs is one of the most dynamic changes in response to stress conditions (Kohli et al., 2013). As tissue/cell sensitivity to PGRs also changes during plant development in response to environmental or genetically coded changes (Davies, 2010), it could be supposed that interactions between PGRs, stress-induced responses and genotype-specific PGRs sensitivity coordinate microspore reprogramming and regulate the final efficiency of ME. The analysis of recent findings obtained with the use of modern analytical techniques brought some new insights into hormonal regulation of microspore reprogramming and the initiation of embryogenic development.

The results of extensive analysis of phytohormone content changes after exposure to various ME-inducing stress treatments in anthers of three highly recalcitrant legume species were presented by Lulsdorf et al. (2012). It was revealed that the most common response was increased level of IAA-asparagine, a putative IAA metabolite. Of the various cytokinins, only *c*ZR increased after the application of stressors.

In *B. napus*, the level of various auxin forms depends significantly on the sample source (leaves, flower buds, isolated microspores) and temperature regimes during the growth of donor plants (10°C/18°C). For the first time Dubas et al. (2012, 2013a) showed that IBA prevailed in isolated microspores and its level could be reduced by low temperature. Interestingly, the combination of low temperature and heat shock reversed this effect. IAA level tends to change in a similar manner to IBA, both in responsive and recalcitrant genotypes. Based on these data, it could be concluded that noticeable changes in the level of both auxins forms caused by stress treatments are important for ME.

Similarly, low temperature ME-initiating treatment (3 weeks at 4°C) meaningfully changed PGRs homeostasis in several DH lines of triticale (Żur et al., 2015). Accumulation of IAA, IBA, *c*Z, *c*ZR, and ABA together with a decrease in *t*Z content was observed in all studied genotypes. It was discovered that as result of cold treatment anthers of highly responsive triticale genotypes were characterized by higher concentrations of IBA, *c*Z,* t*Z, *c*ZR, and lower amount of IAA and KN-like compound in comparison with recalcitrant ones. However, the effects of exogenously applied ABA, PCIB and TIBA suggest that none of the studied PGRs acts alone in the determination of embryogenic competency. An important prerequisite for effective ME seems to be a specific PGR homeostasis – lower auxin rate in comparison with cytokinins and ABA, and lower cytokinin/ABA ratio.

Genetically or/and environmentally determined changes in PGR sensitivity at least partially explain the importance of the timing of hormonal treatment/application. For example, Wassom et al. (2001) showed that modification of maize anther culture medium with various PGRs (ABA, GA_3_, ancymidol, or fluridone) was ineffective in comparison to donor plant treatments, where these substances were pipetted into whorls of field-grown plants 3 days before tassel harvest. Similarly, Liu et al. (2002a,b) and Zheng et al. (2003) demonstrated stimulation of ME in wheat by a combination of high temperature tillers pre-treatment with their ‘inducer chemical formulation.’ It seems that altered PGR homeostasis preceding microspore isolation and transfer to *in vitro* culture triggers changes important for effective ME initiation.

## Summary

The data presented above indicate why standard culture medium optimization using the traditional ‘one-factor-at-a-time’ and ‘trial-and-error’ techniques, which require a considerable amount of time and effort, can sometimes be completely ineffective. As a large number of important crop species, cultivars or genotypes still remain highly recalcitrant to ME, only a much more precise recognition of molecular/physiological/metabolomical background that favors the initiation of embryogenic development could bring any substantial progress. As hormonal homeostasis seems to be one of the most important factors determining cell embryogenic competency, only a more comprehensive approach leading to the recognition of the mechanisms controlling the process could break the barrier of ME recalcitrancy.

## Conflict of Interest Statement

The authors declare that the research was conducted in the absence of any commercial or financial relationships that could be construed as a potential conflict of interest.

## Abbreviations

ABA: abscisic acid
ACC: 1-aminocyclopropane-l-carboxylic acid
AVG: aminoethoxyvinylglycine
AZI: 7-azaindole
BAP: 6-benzylaminopurine
BL: brassinolide
BR: brassinosteroid
CPIBA: chlorophenoxyisobutyric acid
DIC: 3,6-dichloro-2-methoxybenzoic acid (dicamba)
2,4-D: 2,4-dichlorophenoxyacetic acid
EBr: 4-epibrassinolide
ETP: Ethephon
GAs: gibberellins
GA3: gibberellic acid
IAA: indole-3-acetic acid
IBA: indole-3-butyric acid
2iP: N6-(2- isopentenyl)-adenine
JA: jasmonic acid
ME: microspore embryogenesis
NAA: naphthalene-1-acetic acid
1-NOA: 1- naphthoxyacetic acid
NPA: N-1-naphthylphthalamic acid
OCPIB: o-chlorophenoxy-isobutyric acid
PAA: phenylacetic acid
PCIB: p-chlorophenoxyisobutyric acid
PGR(s): plant growth regulator(s)
PIC: 4-amino-3,5,6-trichloropicolinic acid (picloram)
TDZ: N-phenyl-N′-1,2,3-thiadiazol-5-ylurea (thidiazuron)
mT: 6-3-hydroxybenzylaminopurine (metatopoline)
KN: N6-furfuryladenine (kinetin)
SA: salicylic acid
TIBA: 2,3,5-triiodobenzoic acid
Z: zeatin
ZR: zeatin riboside
