# Erratum: Current insights into hormonal regulation of microspore embryogenesis

**DOI:** 10.3389/fpls.2015.00569

**Published:** 2015-07-15

**Authors:** Frontiers Production Office

**Affiliations:** Frontiers Production Office, FrontiersLausanne, Switzerland

**Keywords:** crop species, hormonal regulation, microspore embryogenesis, plant growth regulators, phytohormone crosstalk

Reason for Erratum:

In the original article 2 of the references were cited incorrectly for the name of Teixeira da Silva, J. A. Below are the correct citations:

Ahmadi, B., Alizadeh, K., and Teixeira da Silva, J. A. (2012). Enhanced regeneration of haploid plantlets from microspores of Brassica napus L. using bleomycin, PCIB, and phytohormones. *Plant Cell Tissue Organ Cult*. 109, 525–533. doi: 10.1007/s11240-012-0119-8

Ahmadi, B., Shariatpanahi, M. E., and Teixeira da Silva, J. A. (2014). Efficient induction of microspore embryogenesis using abscisic acid, jasmonic acid and salicylic acid in *Brassica napus* L. *Plant Cell Tissue Organ Cult*. 116, 343–351. doi: 10.1007/s11240-013-0408-x

This error does not change the scientific conclusions of the article in any way.

The original article has been updated.

