# Supplementary material for: Current insights into hormonal regulation of microspore embryogenesis
Source: Front Plant Sci. 2015 Jun 10;6:424. doi: 10.3389/fpls.2015.00424 (PMC4462098; doi:10.3389/fpls.2015.00424)
Supplement: Supplementary file 1 [file Table_1.DOCX]

**Supplementary Material: Table 1S.** Hormonal composition of culture media tested for the most important crop plant species arranged in chronological order. Only the media with new PGRs/PGR combinations or concentration ranges are listed with the concentration units originally used. Culture type (anther culture, AC; isolated microspore culture, MC) and culture phase (pretreatment, Pre; induction, Ind; regeneration, Reg) are specified.

| **Method/phase: PGRs composition of culture medium** | *References* |
| --- | --- |
| ***Hordeum vulgare* (L.)** | |
| AC/Ind: 0.5 mg l^-1^ KN + 2 mg l^-1^ 2,4-D  AC/Reg: 0.5 mg l^-1^ KN + 2 mg l^-1^ IBA →1 mg l^-1^ ZR + 1 mg l^-1^ IBA | *(*[*Kao et al., 1991*](#_ENREF_30)*)* |
| AC, MC/Ind: 2-100 mg l^-1^ PAA  AC, MC/Reg: PGRs free medium | *(*[*Ziauddin et al., 1992*](#_ENREF_78)*)* |
| AC/Ind: 1 mg l^-1^ PAA + 0.2 mg l^-1^ KN ± 10 mg l^-1^ACC/AgNO_3_/STS  AC/Reg: 0.4 mg l^-1^ BAP ± 10 mg l^-1^ ACC/AgNO_3_/STS | *(*[*Evans and Batty, 1994*](#_ENREF_14)*)* |
| AC, MC/Ind: 1-100 µM 2,4-D ± 4 µM BAP  AC, MC/Reg: 0.4 M KN | *(*[*Hoekstra et al., 1996*](#_ENREF_18)*)* |
| AC/Pre: 0.1 µM ABA for 1-4 days/ 40 mg l^-1^ fluridone for 24 hrs  AC/Ind: 1 mg l^-1^ BAP  AC/Reg: PGRs free medium | *(*[*Hoekstra et al., 1997*](#_ENREF_19)*)* |
| AC/Ind: 1 mg l^-1^ BAP ± 0.05 mg l^−1^ ABA ± 0.01 mg l^−1^ TDZ ± 2 mg l^−1^ PAA  AC/Reg: PGRs free medium | *(*[*Ouedraogo et al., 1998*](#_ENREF_46)*)* |
| AC/Ind: 4.4 µM BAP ± 4.5 µM 2,4-D ± 2-4 µM TIBA  AC/Reg: 2.9 µM IAA + 4.4 µM BAP | *(*[*Cistue et al., 1999*](#_ENREF_9)*)* |
| AC, MC/Ind: PGRs-free  AC, MC/Reg: 0.5 mg l^-1^ IAA + 1 mg l^-1^ BAP | *(*[*Castillo et al., 2000*](#_ENREF_7)*)* |
| MC/Ind: 10 mg l^-1^ PAA + 1 mg l^-1^ BAP  MC/Reg: 1 mg l^-1^ BAP | *(*[*Kasha et al., 2001*](#_ENREF_32)*)* |
| AC/Ind: 2 mg l^-1^ NAA + 1 mg l^-1^ BAP  AC/Reg: 0.4 mg l^-1^ NAA + 0.4 mg l^-1^ BAP | *(*[*Jacquard et al., 2003*](#_ENREF_27)*)* |
| MC/Ind: 1 mg l^-1^ BAP (ov)  MC/Reg: 0.2 mg l^-1^ BAP + 0.2 mg l^-1^ IAA | *(*[*Li and Devaux, 2003*](#_ENREF_40)*)* |
| MC/Ind: 1 mg l^-1^ BAP + 1 mg l^-1^ IAA  MC/Reg: PGRs free medium | *(*[*Davies, 2003*](#_ENREF_11)*)* |
| AC/Ind: 2 mg l^-1^ IAA + 1 mg l^-1^ BAP  AC/Reg: 0.4 mg l^-1^ IAA + 0.4 mg l^-1^ BAP | *(*[*Jacquard et al., 2009*](#_ENREF_26)*)* |
| MC/Ind: 1 mg l^-1^ BAP/0.1-1 mg l^-1^ TDZ ± 0.1-1 mg l^-1^ DIC  MC/Reg: 0.2 mg l^-1^ IAA + 0.2 mg l^-1^ BAP /0.1 mg l^-1^ IAA + 0.1 mg l^-1^ mT | *(*[*Esteves et al., 2014*](#_ENREF_13)*)* |
| ***Triticum aestivum* (L.)** | |
| AC/Ind: 1.5 mg l^-1^ 2,4-D + 0.5 mg l^-1^ KN  AC/Reg: 1 mg l^-1^ 2,4-D + 1 mg l^-1^ NAA + 0.1 mg l^-1^ 2iP | *(*[*Schaeffer et al., 1979*](#_ENREF_61)*)* |
| AC/Ind: 1.5 mg l^-1^ 2,4-D + 0.5 mg l^-1^ KN  AC/Reg: 0.5 mg l^-1^ NAA + 0.5 mg l^-1^ KN | *(*[*Sagi and Barnabas, 1989*](#_ENREF_59)*)* |
| AC/Ind: 100 mg l^-1^ PAA/8 mg l^-1^ 2,4-D →100 mg l^-1^ PAA/8 mg l^-1^ 2,4-D + 1 mg l^-1^ IAA  AC/Reg: 0.25 mg l^-1^ BAP | *(*[*Ziauddin et al., 1992*](#_ENREF_78)*)* |
| AC/Ind: 2-8 mg l^-1^ 2,4-D/0.5 mg l^-1^ ZR/0.0001 mg l^-1^ 2iP → 1-1.5 mg l^-1^ IAA/0.001 mg l^-1^ GA_3_/0.0001 mg 2iP/0.001 l^-1^ ABA  AC/Reg: PGRs-free medium | *(*[*Trottier et al., 1993*](#_ENREF_68)*)* |
| MC/Ind:1-10 mg l^-1^ PAA  MC/Reg: 1 mg l^-1^ IAA | *(*[*Hu et al., 1995*](#_ENREF_21)*)* |
| AC/Ind: 2-4 mg l^-1^ PAA + 0.5 mg l^-1^ KN (ov)  AC/Reg: PGRs-free medium | *(*[*Hu and Kasha, 1997*](#_ENREF_20)*)* |
| AC/Ind: 0.5-4 mg l^-1^ 2,4-D →0.2-0.5 mg l^-1^ 2,4-D  AC/Reg: PGRs free medium | *(*[*Zheng and Konzak, 1999*](#_ENREF_74)*)* |
| AC/Ind: 2.0 mg l^-1^ 2.4-D + 0.5 mg l^-1^ KN  AC/Reg: PGRs-free medium | *(*[*Puolimatka and Pauk, 2000*](#_ENREF_53)*)* |
| MC/Ind: 0.2 mg l^-1^ 2,4-D + 0.2 mg l^-1^ KN + 1 mg l^-1^ PAA (ov)  MC/Reg: PGRs-free medium | *(*[*Zheng et al., 2001*](#_ENREF_75)*)* |
| MC/Pre: 10 mg l^-1^ 2,4-D + 2 mg l^-1^ BAP + 3 mg l^-1^ GA_3_ (ov)  MC/Ind, Reg: PGRs- free medium (± ov) | *(*[*Liu et al., 2002*](#_ENREF_42)*)* |
| MC/Ind: 2 mg l^-1^ PAA + 0.5 mg l^-1^ KN  MC/Reg: 0.2 mg l^-1^ PAA + 0.5 mg l^-1^ KN | *(*[*Kasha et al., 2003*](#_ENREF_31)*)* |
| MC/Pre: 0.5 mg l^-1^ KN  MC/Ind: 0.2 mg l^-1^ KN + 0.2 mg l^-1^ 2,4-D + 1 mg l^-1^ PAA (ov)  MC/Reg: PGRs-free medium | *(*[*Zheng et al., 2003*](#_ENREF_76)*)* |
| MC/Ind: 1-4 mg l^-1^ PAA/0.5 mg l^-1^ 2,4-D (ov)  MC/Reg: 1-4 mg l^-1^ PAA/0.5 mg l^-1^ 2,4-D (± ov) | *(*[*Patel et al., 2004*](#_ENREF_47)*)* |
| AC/Ind: 0.5 mg l^-1^ KN + 2 mg l^-1^ 2,4-D  AC/Reg: PGRs-free medium | *(*[*Broughton, 2008*](#_ENREF_6)*)* |
| ***Triticum turgidum* (L.)** | |
| AC/Ind: 2 mg l^-1^ 2,4-D ± 0.5 mg l^-1^ KN  AC/Reg: 0.5-2 mg l^-1^ IAA/0.5 mg l^-1^ NAA ± 1 mg l^-1^ BAP/0.5-1 mg l^-1^ KN/ 0.5 mg l^-1^ Z ± 1-3.5 mg l^-1^ GA_3_ | *(*[*Saidi et al., 1997*](#_ENREF_60)*)* |
| AC/Ind: 8 mg l^-1^ 2,4-D  AC/Reg: 2 mg l^-1^ KN+1 mg l^-1^ IAA | *(*[*Jauhar, 2003*](#_ENREF_28)*)* |
| MC/Ind: 1 mg l^-1^ 2,4-D + 1 mg l^-1^ BAP/KN (ov)  MC/Reg: no data | *(*[*Cistue et al., 2006*](#_ENREF_10)*)* |
| MC/Ind: 0.5 mg l^-1^ KN + 0.5 mg l^-1^ 2,4-D (ov)  MC/Reg: PGRs-free medium | *(*[*Ayed et al., 2010*](#_ENREF_4)*)* |
| ***Zea mays*** **(L.)** | |
| MC/Ind: 0.1 mg l^-1^ TIBA → 2.5 mg l^-1^ DIC + 0.1 mg l^-1^ 2,4-D  MC/Reg: PGRs free medium | *(*[*Pescitelli et al., 1989*](#_ENREF_50)*)* |
| AC/Ind: 0.1-100 *μ*M CPIBA/ 0.1-100 *μ*M qurcetin/ 0.1-100 *μ*M dopamine  AC/Reg: no data | *(*[*Delalonde and Coumans, 1998*](#_ENREF_12)*)* |
| AC/Pre: 50-250 *μ*M ABA/50-250 *μ*M GA_3_/50 *μ*M ancymidol/50 *μ*M fluridone  AC/Ind: 0.3-1 *μ*M AeBA/0.3-1 *μ*M GA_3_/10 *μ*M ancymidol/10 *μ*M fluridone  AC/Reg: no data | *(*[*Wassom et al., 2001*](#_ENREF_70)*)* |
| MC/Ind: 1.2 mg l^-1^ 2,4-D + 1 mg l^-1^ PAA + 0.4 mg l^-1^ KN  MC/Reg: 1 mg l^-1^ NAA + 2 mg l^-1^ KN + 2 mg l^-1^ BAP | *(*[*Zheng et al., 2003*](#_ENREF_76)*)* |
| AC/Ind: 0.1 mg l^-1^ TIBA  AC/Reg: 0.5 mg l^-1^ NAA + 1 mg l^-1^ KN | *(*[*Barnabás, 2003*](#_ENREF_5)*)* |
| ***Oryza sativa* (L.)** | |
| AC/Ind: 2 mg l^-1^ NAA/2,4-D/DIC ± 0.07 mg l^-1^ PIC ± 0.5 mg l^-1^ KN/  2-100 mg l^-1^ PAA/2,4-D + 0.1 mg l^-1^ Z/0.5-1 mg l^-1^ KN  AC/Reg: 1 mg l^-1^ NAA + 4 mg l^-1^ KN | *(*[*Lentini et al., 1995*](#_ENREF_38)*)* |
| MC/Ind: 0.5 mg l^-1^ 2,4-D + 2.5 mg l^-1^ NAA + 0.5 mg l^-1^ KN  MC/Reg: 1 mg l^-1^ NAA + 0.5 mg l^-1^ KN + 2 mg l^-1^ BAP | *(*[*Raina and Irfan, 1998*](#_ENREF_54)*)* |
| AC/Ind: 2-10 mg l^-1^ ABA  AC/Reg: 0.5-1 mg l^-1^ NAA ± 1-8 mg l^-1^ KN ± 1-2 mg l^-1^ BAP | *(*[*Guzman and Arias, 2000*](#_ENREF_17)*)* |
| AC/Ind: 6.79 *μ*M 2,4-D + 5.37 *μ*M NAA + 2.32 *μ*M KN  AC/Reg: 5.71 *μ*M IAA + 9.29 *μ*M KN | *(*[*Trejo-Tapia et al., 2002*](#_ENREF_67)*)* |
| AC/Ind: 1 mg l^-1^ 2,4-D + 0.5 mg l^-1^ BAP + 0.5 mg l^-1^ IAA  AC/Reg: 0.5-4 mg l^-1^ KN | *(*[*Zapata-Arias, 2003*](#_ENREF_73)*)* |
| ***Secale cereale* (L.)** | |
| AC/Ind: 9.05 *μ*M 2,4-D + 2.32 *μ*M KN/ 12.42 *μ*M PIC  AC/Reg: 2.32 *μ*M KN/13.58 *μ*M 2,4-D | *(*[*Rakoczy-Trojanowska et al., 1997*](#_ENREF_55)*)* |
| AC/Ind: 4.5-9 *μ*M DIC/2,4-D ± 2.3 *μ*M KN  AC/Reg: 0.27 *μ*M NAA + 2.22 *μ*M BAP | *(*[*Immonen and Anttila, 1999*](#_ENREF_23)*)* |
| AC/Ind: 0.2 mg l^-1^ 2,4-D + 1 mg l^-1^ KN  AC/Reg: 0.5 mg l^-1^ IAA+1 mg l^-1^ KN | *(*[*Immonen and Tenhola-Roininen, 2003*](#_ENREF_25)*)* |
| MC/Ind: 1.5 mg l^-1^ 2,4-D + 0.5 mg l^-1^ KN  MC/Reg: 0.5 mg l^-1^ 2,4-D + 0.5 mg l^-1^ BAP | *(*[*Pulli and Guo, 2003*](#_ENREF_52)*)* |
| AC, MC/Ind: 2 mg l^-1^ 2,4-D + 0.5 mg l^-1^ KN  AC, MC/Reg; 0.05 mg l^-1^ NAA + 0.5 mg l^-1^ BAP/ 3-5 mg l^-1^ BAP/ 1 mg l^-1^ NAA + 1 mg l^-1^ Z | *(*[*Ma et al., 2004*](#_ENREF_43)*)* |
| ***× Triticosecale*** **Wittm.** | |
| AC/Ind: 0-2 mg l^-1^ 2,4-D + 1 mg l^-1^ IBA  AC/Reg: 1 mg l^-1^ IAA + 1 mg l^-1^ KN | *(*[*Sozinov et al., 1981*](#_ENREF_64)*)* |
| AC/Ind: 2 mg l^-1^ 2,4-D  AC/Reg: 0.4 mg l^-1^ NAA + 1 mg l^-1^ IAA | *(*[*Gonzalez et al., 1997*](#_ENREF_16)*)* |
| AC/Ind: 9 *μ*M 2,4-D + 2.3 *μ*M KN  AC/Reg: 2.9 *μ*M IAA + 4.6 *μ*M KN | *(*[*Immonen and Anttila, 2000*](#_ENREF_24)*)* |
| MC/Ind: 1.5 mg l^-1^ 2,4-D + 0.5 mg l^-1^ KN/ 10 mg l^-1^ PAA/ PGRs free medium  MC/Reg: 0.5 mg l^-1^ NAA + 0.5 mg l^-1^ KN | *(*[*Pauk et al., 2000*](#_ENREF_48)*)* |
| AC/Ind: 1.5 mg l^-1^ 2,4-D + 0.5 mg l^-1^ KN  AC/Reg: PGRs-free medium | *(*[*Tuvesson et al., 2003*](#_ENREF_69)*)* |
| AC/Ind: 2 mg l^-1^ 2,4-D + 0.5 mg l^-1^ KN  AC/Reg: 0.5 mg l^-1^ NAA + 0.5 mg l^-1^ KN | *(*[*Wędzony, 2003*](#_ENREF_72)*)* |
| ***Avena sativa* (L.)** | |
| AC/Ind: PGRs-free/ 1 mg l^-1^ 2,4-D  AC/Reg: 2 mg l^-1^ Z + 0.1-1 mg l^-1^ TIBA | *(*[*Kiviharju et al., 1997*](#_ENREF_34)*)* |
| AC/Ind: 0-8 mg l^-1^ 2,4-D ± 0.5-2 mg l^–1^ KN  AC/Reg: 0-5 mg l^-1^ 2,4-D + 0-5 mg l^-1^ KIN ± 1 mg l^-1^ TIBA ± 1 mg l^-1^ NPA ± 20 mg l^-1^ AgNO_3_ | *(*[*Kiviharju and Tauriainen, 1999*](#_ENREF_35)*)* |
| AC/Ind: 5 mg l^-1^ 2,4-D + 0.5 mg l^–1^ BAP + 20 mg l^-1^ ETP  AC/Reg: 2 mg l^–1^ NAA + 0.5 mg l^–1^ KN | *(*[*Kiviharju et al., 2005*](#_ENREF_33)*)* |
| MC/Ind: 1 mg l^-1^ IAA + 1 mg l^-1^ BAP (CM)  MC/Reg: PGRs free medium | *(*[*Sidhu and Davies, 2009*](#_ENREF_62)*)* |
| AC/Pre: 2 mg l^-1^ 2,4-D  AC/Ind: 5 mg l^-1^ 2,4-D + 0.5 mg l^-1^ BAP + 20 mg l^-1^ ETP  AC/Reg: 0.5 mg l^–1^ NAA + 0.5 mg l^–1^ KN | *(*[*Ponitka and Ślusarkiewicz-Jarzina, 2009*](#_ENREF_51)*)* |
| ***Sorghum* ssp**. | |
| AC/Ind: 0.5-3 mg l^-1^ 2,4-D + 0.5 mg l^-1^ KN  AC/Reg: 0.5 mg l^-1^ KN/ 5 mg l^-1^ NAA/ 2 mg l^-1^ BAP + 1 mg l^-1^ IAA | *(*[*Rose et al., 1986*](#_ENREF_58)*)* |
| AC/Ind: 2-3 mg l^-1^ 2,4-D/1 mg l^-1^ NAA + 0.3-1.5 mg l^-1^ KN ± 2.2 mg l^-1^ Z  AC/Reg: 0.5-3.5 mg l^-1^IAA + 0.5-2.5 mg l^-1^ KN ± 0.5 mg l^-1^ NAA | *(*[*Wen et al., 1991*](#_ENREF_71)*)* |
| AC/Ind: 0.5-2.5 mg l^-1^ 2,4-D  AC/Reg: 1.5-2.5 mg l^-1^ BAP + 0.3 mg l^-1^ IAA | *(*[*Kumaravadivel and Rangasamy, 1994*](#_ENREF_37)*)* |
| AC/Ind: 3.0 mg l^-1^ 2,4-D + 0.3-2.5 mg l^-1^ KN/ 2-2.2 mg l^-1^ Z  AC/Reg: 2 mg l^-1^ IAA + 2.5 mg l^-1^ KN | *(*[*Nakamura et al., 1997*](#_ENREF_44)*)* |
| ***Brassica napus* (L.)** | |
| MC/Ind: 0.5 mg l^-1^ NAA + 0.05 mg l^-1^ BAP  MC/Reg: 0.5 mg l^-1^ NAA + 5 mg l^-1^ BAP | *(*[*Lichter, 1982*](#_ENREF_41)*)* |
| MC/Ind: 0.5 mg l^-1^ NAA  MC/Reg: 0.1% GA_3_ | *(*[*Chuong et al., 1988*](#_ENREF_8)*)* |
| MC/Ind: PGRs-free medium  MC/Reg: ± 3 mg l^-1^ ABA | *(*[*Huang et al., 1991*](#_ENREF_22)*)* |
| MC/Ind: PGRs-free medium  MC/Reg: 0.1 mg l^-1^ GA_3_ | *(*[*Zhou et al., 2002*](#_ENREF_77)*)* |
| MC/Ind: 0.01-100 μM EBr/ 0.1-1 μM BL  MC/Reg: PGRs-free medium | *(*[*Ferrie et al., 2005*](#_ENREF_15)*)* |
| MC/Ind: 0.1-10 μM AVG/ 2.5-25 μM CoCl_2_/ 0.1-10 μM STS/ 10-200 μM SAM/ 10-100 μM ACC/ 10-100 μM ETP  MC/Reg: no data | *(*[*Leroux et al., 2009*](#_ENREF_39)*)* |
| MC/Ind: 15-45 mg l^-1^ 2,4-D for 15-40 min  MC/Reg: 0.01 mg l^-1^ GA_3_ | *(*[*Ardebili et al., 2011*](#_ENREF_3)*)* |
| MC/Ind: 1-5 mg l^-1^ PCIB for 12-24 hrs  MC/Reg: 0.05 mg l^-1^ GA_3_/0.1 mg l^-1^BAP + 0.2 mg l^-1^ IAA | *(*[*Ahmadi et al., 2012*](#_ENREF_1)*)* |
| MC/Ind: 0.2-5 mg l^-1^ ABA/JA/SA for 6-24 hrs  MC/Reg: 0.1 mg l^-1^ GA_3_ | *(*[*Ahmadi et al., 2014*](#_ENREF_2)*)* |
| ***Solanum tuberosum* (L.)** | |
| AC/Ind: 1 mg l^-1^ BAP/2 mg l^-1^ 2,4-D/2 mg l^-1^ 2,4-D + 0.1 mg l^-1^ ZR  AC/Reg: no data | *(*[*Říhová and Tupý, 1996*](#_ENREF_56)*)* |
| AC/Ind: 0.1 mg l^-1^ IAA+1-3 mg l^-1^ BAP  AC/Reg: 0.1 mg l^-1^ GA_3_ | *(*[*Tai and Xiong, 2003*](#_ENREF_65)*)* |
| ***Glycine max* (L.)** | |
| AC/Ind: 2 mg l^-1^ 2,4-D + 0.5 mg l^-1^ BAP/0.5 mg l^-1^ KN/ 1 mg l^-1^ 2,4-D + 0.5 mg l^-1^ KN  AC/Reg: no data | *(*[*Kaltchuk-Santos et al., 1997*](#_ENREF_29)*)* |
| AC/Ind: 2-10 mg l^-1^ 2,4-D + 0.5 mg l^-1^ BAP → 2 mg l^-1^ 2,4-D  AC/Reg: no data | *(*[*Rodrigues et al., 2004*](#_ENREF_57)*)* |
| AC/Ind: 2 mg l^-1^ 2,4-D + 0.5 mg l^-1^ IBA/BAP/KN ± 1 mg l^-1^ NAA  AC/Reg: 0.4 mg l^-1^ NAA+ 0.4 mg l^-1^ BAP | *(*[*Tiwari et al., 2004*](#_ENREF_66)*)* |
| ***Lupinus* ssp.** | |
| AC/Ind: 2.26 μM 2,4-D + 4.65 μM KIN + 2.22 μM BAP  AC/Reg: PGRs free medium | *(*[*Ormerod and Caligari, 1994*](#_ENREF_45)*)* |
| AC/Ind: 1.0 mg l^-1^ NAA + 1.0 mg l^-1^ BAP  AC/Reg: no data | *(*[*Skrzypek et al., 2008*](#_ENREF_63)*)* |
| AC/Ind: 2-10 mg l^-1^ 2,4 D + 0.5 mg l^-1^ KN/BAP/NAA  AC/Reg: 0.5 mg l^-1^ NAA + 2 mg l^-1^ BAP | *(*[*Kozak et al., 2012*](#_ENREF_36)*)* |
| ***Manihot esculenta*** | |
| AC/Ind: 8 mg l^− 1^ 2,4-D → 2 mg l^− 1^ 2,4-D  AC/Reg: PGRs-free medium → 2 mg l^− 1^ BAP + 0.5 mg l^− 1^ NAA → 0.5 mg l^− 1^ BAP | *(*[*Perera et al., 2014*](#_ENREF_49)*)* |

CM – conditioned medium; (ov) – ovary co-culture;

ACC – 1-aminocyclopropane-1-carboxylic acid; AVG – aminoethoxyvinylglycine; AZI – 7-azaindole; BL – brassinolide; DIC – dicamba; EBr – 4-epibrassinolide; ETP – Ethephon; PIC – picloram; TDZ – thidiazuron; mT – meta-topoline; KN – kinetin; SAM – S-adenosyl-methionine; STS – silver thiosulphate (Ag_2_S_2_O_3_); Z – zeatin; ZR – zeatin riboside; 2iP – isopentenyladenine

“→” – transfer; “/” – substitute; “±” – with or without

References

Ahmadi, B., Alizadeh, K., and Teixeira Da Silva, J. (2012). Enhanced regeneration of haploid plantlets from microspores of *Brassica napus* L. using bleomycin, PCIB, and phytohormones. *Plant Cell, Tissue and Organ Culture* 109**,** 525-533. doi: 10.1007/s11240-012-0119-8.

Ahmadi, B., Shariatpanahi, M.E., and Da Silva, J.a.T. (2014). Efficient induction of microspore embryogenesis using abscisic acid, jasmonic acid and salicylic acid in *Brassica napus* L. *Plant Cell, Tissue and Organ Culture* 116**,** 343-351. doi: 10.1007/s11240-013-0408-x.

Ardebili, S.H., Shariatpanahi, M.E., Amiri, R., Emamifar, M., Oroojloo, M., Nematzadeh, G., Noori, S.a.S., and Heberle-Bors, E. (2011). Effect of 2,4-D as a Novel Inducer of Embryogenesis in Microspores of *Brassica napus* L. *Czech Journal of Genetics and Plant Breeding* 47**,** 114-122.

Ayed, O.S., De Buyser, J., Picard, E., Trifa, Y., and Amara, H.S. (2010). Effect of pre-treatment on isolated microspores culture ability in durum wheat (*Triticum turgidum* subsp. *durum* Desf.). *Journal of Plant Breeding and Crop Science* 2**,** 30-38.

Barnabás, B. (2003). "Anther culture of maize (*Zea mays* L.)," in *Doubled Haploid Production in Crop Plants,* eds. M. Maluszynski, K.J. Kasha, B.P. Forster & I. Szarejko. (Drdrecht/Boston/London: Springer Netherlands), 103-108.

Broughton, S. (2008). Ovary co-culture improves embryo and green plant production in anther culture of Australian spring wheat (*Triticum aestivum* L.). *Plant Cell Tissue and Organ Culture* 95**,** 185-195. doi: 10.1007/s11240-008-9432-7.

Castillo, A.M., Vallés, M.P., and Cistué, L. (2000). Comparison of anther and isolated microspore cultures in barley. Effects of culture density and regeneration medium. *Euphytica* 113**,** 1-8. doi: 10.1023/A:1003937530907.

Chuong, P.V., Pauls, K.P., and Beversdorf, W.D. (1988). High-frequency embryogenesis in male sterile plants of *Brassica napus* through microspore culture. *Canadian Journal of Botany-Revue Canadienne De Botanique* 66**,** 1676-1680.

Cistue, L., Ramos, A., and Castillo, A.M. (1999). Influence of anther pretreatment and culture medium composition on the production of barley doubled haploids from model and low responding cultivars. *Plant Cell Tissue and Organ Culture* 55**,** 159-166. doi: 10.1023/a:1006130028396.

Cistue, L., Soriano, M., Castillo, A.M., Valles, M.P., Sanz, J.M., and Echavarri, B. (2006). Production of doubled haploids in durum wheat (*Triticum turgidum* L.) through isolated microspore culture. *Plant Cell Reports* 25**,** 257-264. doi: 10.1007/s00299-005-0047-8.

Davies, P.A. (2003). "Barley isolated microspore culture (IMC) method " in *Doubled Haploid Production in Crop Plants,* eds. M. Maluszynski, K.J. Kasha, B.P. Forster & I. Szarejko. (Drdrecht/Boston/London: Springer Netherlands), 49-52.

Delalonde, M., and Coumans, M. (1998). Effect of IAA content Modulators on peroxidase activity and on endogenous IAA during cold pretreatment of maize anthers prior to androgenesis. *Plant Growth Regulation* 26**,** 123-130. doi: 10.1023/A:1006131620264.

Esteves, P., Clermont, I., Marchand, S., and Belzile, F. (2014). Improving the efficiency of isolated microspore culture in six-row spring barley: II-exploring novel growth regulators to maximize embryogenesis and reduce albinism. *Plant Cell Reports* 33**,** 871-879. doi: 10.1007/s00299-014-1563-1.

Evans, J., and Batty, N. (1994). Ethylene precursors and antagonists increase embryogenesis of *Hordeum vulgare* L. anther culture. *Plant Cell Reports* 13**,** 676-678. doi: 10.1007/BF00231622.

Ferrie, A.M.R., Dirpaul, J., Krishna, P., Krochko, J., and Keller, W.A. (2005). Effects of brassinosteroids on microspore embryogenesis in *Brassica* species. *In Vitro Cellular & Developmental Biology-Plant* 41**,** 742-745. doi: 10.1079/ivp2005690.

Gonzalez, M., Hernandez, I., and Jouve, N. (1997). Analysis of anther culture response in hexaploid triticale. *Plant Breeding* 116**,** 302-304. doi: 10.1111/j.1439-0523.1997.tb01003.x.

Guzman, M., and Arias, F.J.Z. (2000). Increasing anther culture efficiency in rice (*Oryza sativa* L.) using anthers from ratooned plants. *Plant Science* 151**,** 107-114. doi: 10.1016/s0168-9452(99)00204-6.

Hoekstra, S., Hoekstra, S., Hoekstra, I.R., Hoekstra, R.A., and Hoekstra, E. (1996). The Interaction of 2,4-D Application and Mannitol Pretreatment in Anther and Microspore Culture of *Hordeum vulgare* L. cv. Igri. *Journal of Plant Physiology* 148**,** 696-700. doi: 10.1016/S0176-1617(96)80370-1.

Hoekstra, S., Vanbergen, S., Vanbrouwershaven, I.R., Schilperoort, R.A., and Wang, M. (1997). Androgenesis in *Hordeum vulgare* L: Effects of mannitol, calcium and abscisic acid on anther pretreatment. *Plant Science* 126**,** 211-218. doi: 10.1016/s0168-9452(97)00096-4.

Hu, T., and Kasha, K.J. (1997). Improvement of isolated microspore culture of wheat (*Triticum aestivum* L) through ovary co-culture. *Plant Cell Reports* 16**,** 520-525.

Hu, T.C., Ziauddin, A., Simion, E., and Kasha, K.J. (1995). Isolated microspore culture of wheat (*Triticum aestivum* L.) in a defined media I. Effects of pretreatment, isolation methods, and hormones. *In Vitro Cellular & Developmental Biology - Plant* 31**,** 79-83. doi: 10.1007/BF02632241.

Huang, B., Bird, S., Kemble, R., Miki, B., and Keller, W. (1991). Plant regeneration from microspore-derived embryos of *Brassica napus*: Effect of embryo age, culture temperature, osmotic pressure, and abscisic acid. *In Vitro Cellular & Developmental Biology - Plant* 27**,** 28-31. doi: 10.1007/BF02632058.

Immonen, S., and Anttila, H. (1999). Cold pretreatment to enhance green plant regeneration from rye anther culture. *Plant Cell Tissue and Organ Culture* 57**,** 121-127. doi: 10.1023/a:1006381516632.

Immonen, S., and Anttila, H. (2000). Media Composition and Anther Plating for Production of Androgenetic Green Plants from Cultivated Rye (*Secale cereale* L.). *Journal of Plant Physiology* 156**,** 204-210. doi: 10.1016/S0176-1617(00)80307-7.

Immonen, S., and Tenhola-Roininen, T. (2003). "Protocol for rye anther culture," in *Doubled Haploid Production in Crop Plants,* eds. M. Maluszynski, K.J. Kasha, B.P. Forster & I. Szarejko. (Drdrecht/Boston/London: Springer Netherlands), 141-150.

Jacquard, C., Mazeyrat-Gourbeyre, F., Devaux, P., Boutilier, K., Baillieul, F., and Clement, C. (2009). Microspore embryogenesis in barley: anther pre-treatment stimulates plant defence gene expression. *Planta* 229**,** 393-402. doi: 10.1007/s00425-008-0838-6.

Jacquard, C., Wojnarowiez, G., and Clement, C. (2003). "Anther culture in barley," in *Doubled Haploid Production in Crop Plants,* eds. M. Maluszynski, K.J. Kasha, B.P. Forster & I. Szarejko. (Drdrecht/Boston/London: Springer Netherlands), 21-28.

Jauhar, P.P. (2003). Formation of 2n gametes in durum wheat haploids: Sexual polyploidization. *Euphytica* 133**,** 81-94. doi: 10.1023/a:1025692422665.

Kaltchuk-Santos, E., Mariath, J., Mundstock, E., Hu, C.-Y., and Bodanese-Zanettini, M. (1997). Cytological analysis of early microspore divisions and embryo formation in cultured soybean anthers. *Plant Cell, Tissue and Organ Culture* 49**,** 107-115. doi: 10.1023/A:1005897915415.

Kao, K.N., Saleem, M., Abrams, S., Pedras, M., Horn, D., and Mallard, C. (1991). Culture conditions for induction of green plants from barley microspores by anther culture methods. *Plant Cell Reports* 9**,** 595-601.

Kasha, K.J., Simion, E., Miner, M., Letarte, J., Hu, T.C., and Wojnarowiez, G. (2003). "Haploid whea tisolated microspore culture protocol," in *Doubled Haploid Production in Crop Plants,* eds. M. Maluszynski, K.J. Kasha, B.P. Forster & I. Szarejko. (Drdrecht/Boston/London: Springer Netherlands), 77-82.

Kasha, K.J., Simion, E., Oro, R., Yao, Q.A., Hu, T.C., and Carlson, A.R. (2001). An improved *in vitro* technique for isolated microspore culture of barley. *Euphytica* 120**,** 379-385.

Kiviharju, E., Moisander, S., and Laurila, J. (2005). Improved green plant regeneration rates from oat anther culture and the agronomic performance of some DH lines. *Plant Cell, Tissue and Organ Culture* 81**,** 1-9. doi: 10.1007/s11240-004-1560-0.

Kiviharju, E., Puolimatka, M., and Pehu, E. (1997). Regeneration of anther-derived plants of *Avena* sterilis. *Plant Cell Tissue and Organ Culture* 48**,** 147-152. doi: 10.1023/a:1005821502596.

Kiviharju, E.M., and Tauriainen, A.A. (1999). 2,4-Dichlorophenoxyacetic acid and kinetin in anther culture of cultivated and wild oats and their interspecific crosses: plant regeneration from *A. sativa* L. *Plant Cell Reports* 18**,** 582-588. doi: 10.1007/s002990050626.

Kozak, K., Galek, R., Waheed, M.T., and Sawicka-Sienkiewicz, E. (2012). Anther culture of *Lupinus angustifolius*: callus formation and the development of multicellular and embryo-like structures. *Plant Growth Regulation* 66**,** 145-153. doi: 10.1007/s10725-011-9638-2.

Kumaravadivel, N., and Rangasamy, S.R.S. (1994). Plant regeneration from sorghum anther cultures and field evaluation of progeny. *Plant Cell Reports* 13**,** 286-290.

Lentini, Z., Reyes, P., Martínez, C.P., and Roca, W.M. (1995). Androgenesis of highly recalcitrant rice genotypes with maltose and silver nitrate. *Plant Science* 110**,** 127-138. doi: 10.1016/0168-9452(95)04180-3.

Leroux, B., Carmoy, N., Giraudet, D., Potin, P., Larher, F., and Bodin, M. (2009). Inhibition of ethylene biosynthesis enhances embryogenesis of cultured microspores of *Brassica napus*. *Plant Biotechnology Reports* 3**,** 347-353. doi: 10.1007/s11816-009-0109-4.

Li, H., and Devaux, P. (2003). High frequency regeneration of barley doubled haploid plants from isolated microspore culture. *Plant Science* 164**,** 379-386. doi: 10.1016/S0168-9452(02)00424-7.

Lichter, R. (1982). Induction of haploid plants from isolated pollen of *Brassica napus*. *Zeitschrift für Pflanzenphysiologie* 105**,** 427-434.

Liu, W., Zheng, M.Y., and Konzak, C.F. (2002). Improving green plant production via isolated microspore culture in bread wheat (*Triticum aestivum* L.). *Plant Cell Reports* 20**,** 821-824. doi: 10.1007/s00299-001-0408-x.

Ma, R., Guo, Y.-D., and Pulli, S. (2004). Comparison of Anther and Microspore Culture in the Embryogenesis and Regeneration of Rye (*Secale cereale*). *Plant Cell, Tissue and Organ Culture* 76**,** 147-157. doi: 10.1023/B:TICU.0000007294.68389.ed.

Nakamura, S., Can, N.D., and Yoshida, T. (1997). Study on callus induction from anther and inflorescense culture of sorghum. *Journal of the Faculty of Agriculture Kyushu University* 42**,** 1-9.

Ormerod, A.J., and Caligari, P.D.S. (1994). Anther and microspore culture of *Lupinus albus* in liquid culture medium. *Plant Cell, Tissue and Organ Culture* 36**,** 227-236. doi: 10.1007/BF00037724.

Ouedraogo, J.T., St-Pierre, C.A., Collin, J., Rioux, S., and Comeau, A. (1998). Effect of amino acids, growth regulators and genotype on androgenesis in barley. *Plant Cell Tissue and Organ Culture* 53**,** 59-66.

Patel, M., Darvey, N., Marshall, D., and Berry, J. (2004). Optimization of culture conditions for improved plant regeneration efficiency from wheat microspore culture. *Euphytica* 140**,** 197-204. doi: 10.1007/s10681-004-3036-z.

Pauk, J., Puolimatka, M., Toth, K.L., and Monostori, T. (2000). *In vitro* androgenesis of triticale in isolated microspore culture. *Plant Cell Tissue and Organ Culture* 61**,** 221-229. doi: 10.1023/a:1006416116366.

Perera, P.I.P., Ordoñez, C.A., Becerra Lopez-Lavalle, L.A., and Dedicova, B. (2014). A milestone in the doubled haploid pathway of cassava. *Protoplasma* 251**,** 233-246.

Pescitelli, S.M., Mitchell, J.C., Jones, A.M., Pareddy, D.R., and Petolino, J.F. (1989). High-frequency androgenesis from isolated microspores of maize. *Plant Cell Reports* 7**,** 673-676.

Ponitka, A., and Ślusarkiewicz-Jarzina, A. (2009). Regeneration of oat androgenic plants in relation to induction media and culture conditions of embryo-like structures. *Acta Societatis Botanicorum Poloniae* 78**,** 209-213.

Pulli, S., and Guo, Y.-D. (2003). "Microspore culture of rye," in *Doubled Haploid Production in Crop Plants,* eds. M. Maluszynski, K.J. Kasha, B.P. Forster & I. Szarejko. (Drdrecht/Boston/London: Springer Netherlands), 151-154.

Puolimatka, M., and Pauk, J. (2000). Effect of Induction Duration and Medium Composition on Plant Regeneration in Wheat (*Triticum aestivum* L.) Anther Culture. *Journal of Plant Physiology* 156**,** 197-203. doi: 10.1016/S0176-1617(00)80306-5.

Raina, S.K., and Irfan, S.T. (1998). High-frequency embryogenesis and plantlet regeneration from isolated microspores of indica rice. *Plant Cell Reports* 17**,** 957-962. doi: 10.1007/s002990050517.

Rakoczy-Trojanowska, M., Smiech, M., and Malepszy, S. (1997). The influence of genotype and medium on rye (*Secale cereale* L) anther culture. *Plant Cell Tissue and Organ Culture* 48**,** 15-21. doi: 10.1023/a:1005792912316.

Říhová, L., and Tupý, J. (1996). Influence of 2,4-d and lactose on pollen embryogenesis in anther culture of potato. *Plant Cell, Tissue and Organ Culture* 45**,** 269-272. doi: 10.1007/BF00043641.

Rodrigues, L.R., Forte, B.D.C., Oliveira, J.M.S., Mariath, J.E.A., and Bodanese-Zanettini, M.H. (2004). Effects of light conditions and 2,4-D concentration in soybean anther culture. *Plant Growth Regulation* 44**,** 125-131. doi: 10.1007/s10725-004-2811-0.

Rose, J.B., Dunwell, J.M., and Sunderland, N. (1986). Anther culture of *Sorghum bicolor* (L) Moench .1. Effect of panicle pretreatment, anther incubation-temperature and 2,4-D concentration. *Plant Cell Tissue and Organ Culture* 6**,** 15-22. doi: 10.1007/bf00037754.

Sagi, L., and Barnabas, B. (1989). Evidence for cytoplasmic control of invitro microspore embryogenesis in the anther culture of wheat (*Triticum aestivum* L.). *Theoretical and Applied Genetics* 78**,** 867-872.

Saidi, N., Cherkaoui, S., Chlyah, A., and Chlyah, H. (1997). Embryo formation and regeneration in *Triticum turgidum* ssp. *durum* anther culture. *Plant Cell, Tissue and Organ Culture* 51**,** 27-33. doi: 10.1023/A:1005765529154.

Schaeffer, G.W., Baenziger, P.S., and Worley, J. (1979). Haploid Plant Development from Anthers and *In Vitro* Embryo Culture of Wheat. *Crop Sci.* 19**,** 697-702. doi: 10.2135/cropsci1979.0011183X001900050038x.

Sidhu, P., and Davies, P. (2009). Regeneration of fertile green plants from oat isolated microspore culture. *Plant Cell Reports* 28**,** 571-577. doi: 10.1007/s00299-009-0684-4.

Skrzypek, E., Czyczyło-Mysza, I., Marcińska, I., and Wędzony, M. (2008). Prospects of androgenetic induction in *Lupinus* spp. *Plant Cell Tissue Organ Culture* 94**,** 131-137.

Sozinov, A., Lukjanjuk, S., and Ignatova, S. (1981). Anther cultivation and induction of haploid plants in *triticale*. *Zeitschrift Fur Pflanzenzuchtung-Journal of Plant Breeding* 86**,** 272-285.

Tai, G.C.C., and Xiong, X.Y. (2003). "Haploid production of potatoes by anther culture," in *Doubled Haploid Production in Crop Plants,* eds. M. Maluszynski, K.J. Kasha, B.P. Forster & I. Szarejko. (Drdrecht/Boston/London: Springer Netherlands), 229-234.

Tiwari, S., Shanker, P., and Tripathi, M. (2004). Effects of genotype and culture medium on i*n vitro* androgenesis in soybean (*Glycine max* Merr.). *Indian Journal of Biotechnology* 3**,** 441-444.

Trejo-Tapia, G., Amaya, U.M., Morales, G.S., Sanchez, A.D., Bonfil, B.M., Rodriguez-Monroy, M., and Jimenez-Aparicio, A. (2002). The effects of cold-pretreatment, auxins and carbon source on anther culture of rice. *Plant Cell Tissue and Organ Culture* 71**,** 41-46. doi: 10.1023/a:1016558025840.

Trottier, M.-C., Collin, J., and Comeau, A. (1993). Comparison of media for their aptitude in wheat anther culture. *Plant Cell, Tissue and Organ Culture* 35**,** 59-67. doi: 10.1007/BF00043940.

Tuvesson, S., Von Post, R., and Ljungberg, A. (2003). "Triticale anther culture," in *Doubled Haploid Production in Crop Plants,* eds. M. Maluszynski, K.J. Kasha, B.P. Forster & I. Szarejko. (Drdrecht/Boston/London: Springer Netherlands), 117-122.

Wassom, J.J., Mei, C., Rocheford, T.R., and Widholm, J.M. (2001). Interaction of environment and ABA and GA treatments on the maize anther culture response. *Plant Cell Tissue and Organ Culture* 64**,** 69-72. doi: 10.1023/a:1010671613695.

Wen, F.S., Sorensen, E.L., Barnett, F.L., and Liang, G.H. (1991). Callus induction and plant regeneration from anther and inflorescence culture of sorghum. *Euphytica* 52**,** 177-181. doi: 10.1007/bf00029394.

Wędzony, M. (2003). "Protocol for anther culture in hexaploid triticale (X *Triticosecale* Wittm.)," in *Doubled Haploid Production in Crop Plants,* eds. M. Maluszynski, K.J. Kasha, B.P. Forster & I. Szarejko. (Drdrecht/Boston/London: Springer Netherlands), 123-128.

Zapata-Arias, F.J. (2003). "Laboratory protocol for anther culture technique in rice," in *Doubled Haploid Production in Crop Plants,* eds. M. Maluszynski, K.J. Kasha, B.P. Forster & I. Szarejko. (Drdrecht/Boston/London: Springer Netherlands), 109-116.

Zheng, M.Y., and Konzak, C.F. (1999). Effect of 2,4-dichlorophenoxyacetic acid on callus induction and plant regeneration in anther culture of wheat (*Triticum aestivum* L.). *Plant Cell Reports* 19**,** 69-73. doi: 10.1007/s002990050712.

Zheng, M.Y., Liu, W., Weng, Y., Polle, E., and Konzak, C.F. (2001). Culture of freshly isolated wheat (*Triticum aestivum* L.) microspores treated with inducer chemicals. *Plant Cell Reports* 20**,** 685-690.

Zheng, M.Y., Liu, W., Weng, Y., Polle, E., and Konzak, C.F. (2003). "Production of doubled haploids in wheat (*Triticum aestivum* L.) through microspore embryogenesis triggered by inducer chemicals," in *Doubled Haploid Production in Crop Plants,* eds. M. Maluszynski, K.J. Kasha, B.P. Forster & I. Szarejko. Springer Netherlands), 83-94.

Zhou, W.J., Tang, G.X., and Hagberg, P. (2002). Efficient production of doubled haploid plants by immediate colchicine treatment of isolated microspores in winter *Brassica napus*. *Plant Growth Regulation* 37**,** 185-192. doi: 10.1023/a:1020561201125.

Ziauddin, A., Marsolais, A., Simion, E., and Kasha, K.J. (1992). Improved plant regeneration from wheat anther and barley microspore culture using phenylacetic acid (PAA). *Plant Cell Reports* 11**,** 489-498. doi: 10.1007/BF00236263.
